# New Methods for Two‐Stage Treatment Switching Estimation

**DOI:** 10.1002/pst.2462

**Published:** 2025-02-04

**Authors:** Dan Jackson, Di Ran, Fanni Zhang, Mario Ouwens, Vitaly Druker, Michael Sweeting, Robert Hettle, Ian R. White

**Affiliations:** ^1^ AstraZeneca Cambridge UK; ^2^ AstraZeneca Gaithersburg Maryland USA; ^3^ AstraZeneca Mölndal Sweden; ^4^ MRC Clinical Trials Unit at UCL London UK

**Keywords:** counterfactuals, crossover, multiple imputations, survival analysis

## Abstract

Treatment switching is common in randomized trials of oncology treatments. For example, control group patients may receive the experimental treatment as a subsequent therapy. One possible estimand is the effect of trial treatment if this type of switching had instead not occurred. Two‐stage estimation is an established approach for estimating this estimand. We argue that other estimands of interest instead describe the effect of trial treatments if the proportion of patients who switched was different. We give precise definitions of such estimands. By motivating estimands using real‐world data, decision‐making in universal health care systems is facilitated. Focusing on estimation, we show that an alternative choice of secondary baseline, the time of first subsequent treatment, is easily defined, and widely applicable, and makes alternative estimands amenable to two‐stage estimation. We develop methodology using propensity scores, to adjust for confounding at a secondary baseline, and a new quantile matching technique that can be used to implement any parametric form of the post‐secondary baseline survival model. Our methodology was motivated by a recent immuno‐oncology trial where a substantial proportion of control group patients subsequently received a form of immunotherapy.

## 1 Introduction

Treatment switching [1, 2] is common in randomized trials of oncology treatments. This occurs when a patient ceases to receive their randomized treatment and instead receives an alternative subsequent therapy. For example, switching to the experimental treatment may be permitted in the control arm in the situation where the experimental treatment has been shown to be effective in interim analyses.

The usual concern when treatment switching occurs is that a treatment policy strategy will not reflect the actual benefit of receiving the experimental treatment. Our overall objective is to define hypothetical estimands that can be used to assess the impact of treatment switching, and then estimate them by performing adjusted analyses. We will therefore compare the results from intention‐to‐treat (ITT) analyses to those from our adjusted analyses. We use the estimands framework from ICH E9(R1) [3] when making this comparison, where estimands have the following attributes: treatment, population, variable (or endpoint), intercurrent events and population summary. We will conceptualise treatment switching as an ‘intercurrent event’ [3] in our adjusted analyses, for which we will adopt several different hypothetical strategies. We describe, and justify, these strategies in Section 3 below. This work was motivated by a recent immuno‐oncology trial where the main concern is that control group patients switch to immunotherapy and so a treatment that is similar, in an important sense, to the two experimental treatments. We will explain below why experimental group patients switching treatments in this way is not, in the context of our example, a source of concern. However, this may be a serious issue in other applications and we return to this in the discussion.

A variety of statistical methods [1, 2, 4, 5, 6] have been developed to adjust for treatment switching. In the context of time‐to‐event data, there are three advanced statistical methodologies that adjust for control group patients switching to the experimental treatment: the rank preserving structural failure time model [7, 8] (RPSFTM), inverse probability censoring weighting [9] (IPCW) and two‐stage estimation [10, 11]. The conventional RPSFTM assumes that there is a common treatment effect (an acceleration factor), that is constant for all patients throughout the trial, where this treatment effect is estimated using g‐estimation. Adjusted analyses can then be performed by removing the treatment benefit (or harm) for control group patients who subsequently receive the experimental treatment. Although different definitions of time on experimental treatment may be used in the analysis, for example, patients may be considered to be on experimental treatment from the time this is first administered or only while receiving this treatment [1], the assumption of a common treatment effect is strong. However, the RPSFTM has the advantage that there is no need to adjust for confounding and it has been enhanced [12] to include an additional acceleration factor associated with an alternative subsequent therapy. IPCW artificially censors patients when they switch and the resulting time‐to‐event data are weighted, based on a model of the probability of being censored. The main difficulty is that this model must be correctly specified [4], or more pragmatically it must be adequate. Hence all important predictors must be used and we assume that there is no unmeasured confounding.

Two‐stage estimation [10, 11] provides our focus and adopts a different approach where a secondary baseline, a timepoint before which treatment switching could not occur [1], is used. Here we use the same methods as in an observational study to model the post‐secondary baseline survival times in the first stage [1] and adjusted analyses are performed by removing the treatment benefit (or harm), in a similar way to the RPSFTM, in the second stage. The flexible and conventional nature of the first stage modelling is an advantage of using two‐stage estimation instead of the RPSFTM. Furthermore, as Latimer et al. [11] point out, the ‘no unmeasured confounders’ assumption is then only required at the secondary baseline and the modelling of the treatment switching process is not needed. Hence the two‐stage approach also has some important advantages over IPCW. In the first stage, we adjust for confounders, where we assume that there is no unmeasured confounding at the secondary baseline [10, 11]. The conventional choice of secondary baseline is the time of disease progression [10, 11], which requires that switching occurs at (or, at least, near) this event. A limitation of conventional two‐stage analyses is that, by removing all treatment benefits (or harms), they only facilitate one type of hypothetical estimand. A fuller narrative, for example, motivated by asking how much further treatment effects may have been affected if the extent of this form of treatment switching had been greater, is facilitated by considering further estimands. We will show how additional estimands can be used to extend the narrative in this way.

Latimer et al. [13] discuss the assumptions required by two‐stage estimation and propose the *TSEgest* approach to relax them. We instead propose using an alternative secondary baseline [14] at the time of the first subsequent treatment. The advantages of our secondary baseline include that it is clearly defined and widely applicable. Another advantage is that we then compare patients who switch to different forms of subsequent treatments, who may be more comparable than patients who do, or do not, switch to the experimental treatment after disease progression.

We build upon the approach of Ouwens et al. [14], by also using the time of the first subsequent treatment as the secondary baseline, and extend this further in a variety of novel ways. For example, one of our proposed estimands is motivated by a real‐world study. This innovative feature of our proposals facilitates a wider narrative about the implications of treatment switching, and so decision‐making in universal health care systems. We develop methodology using propensity scores to adjust for confounding at a secondary baseline, where we clarify the target populations that effects should be estimated. We also propose a new quantile matching technique that can be used to implement any parametric form of the post‐secondary baseline survival model, so that the analyst is not restricted to using accelerated failure time models.

One difficulty presented by two‐stage approaches is the wide variety of choices available to the analyst. For example, many different parametric survival models [15] for post‐secondary baseline survival times may be used, for which bootstrapping [16] may be used to allow for parameter uncertainty. Furthermore, analyses may be performed with, or without, re‐censoring [17, 18]. Different methods for confounder adjustment, for example, propensity score weighting [19] may also be used. We will discuss the most appropriate type of propensity score weighting and explore a wide range of possibilities in our example to illustrate these possibilities. We do not however attempt to exhaust them and we return to this issue in the discussion.

The rest of the article is set out as follows. In Section 2 we present our motivating example, a recent immuno‐oncology trial where a substantial proportion of control group patients subsequently receive a form of immunotherapy. We also examine our choice of secondary baseline in the context of this example. In Section 3 we discuss the different strategies that we will use for handling the intercurrent event, where patients switch to a form of immunotherapy, in defining the estimands. In Section 4 we describe the standard methods for two‐stage estimation, where progression is used as the secondary baseline. In Section 5 we describe our new methods for two‐stage estimation and in Section 6 we illustrate them using our motivating example. We conclude with a discussion in Section 7.

## 2 Motivating Example

We re‐examine the primary overall survival analyses of the MYSTIC trial [20]. This open‐label, phase three clinical trial randomized (1:1:1) patients with metastatic non‐small cell lung cancer to receive either chemotherapy (control group), durvalumab (first experimental immunotherapy treatment) or durvalumab plus tremelimumab (second experimental immunotherapy treatment). See Rizvi et al. [20] for full details of the study design and patient inclusion criteria. We follow Rizvi et al. [20] in restricting the primary analysis population to patients with ≥ 25% of tumor cells expressing PD‐L1. In the analysis of Rizvi et al. this decision resulted in the inclusion of 488 patients. However, we are only able to include 479 of these because of issues relating to patient consent.

### 2.1 ITT Analyses

**FIGURE 1:**
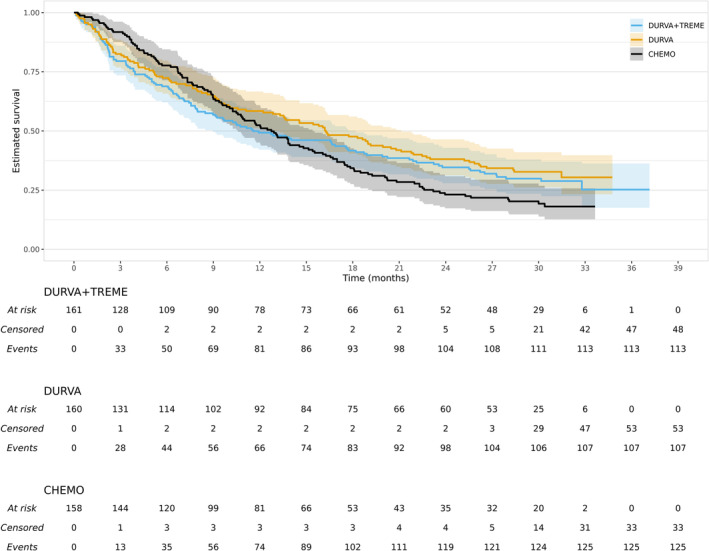
Kaplan–Meier curves for the MYSTIC trial. Durva + treme, durva and chemo refer to the durvalumab plus tremelimumab, durvalumab and chemotherapy groups, respectively.

The primary overall survival analysis results are shown in Rizvi et al.; all our ITT results in this section are very similar but differ slightly because of our need to omit nine patients. Kaplan–Meier (KM) curves are shown for each treatment group in Figure 1, following the proposal of Morris et al. to show an extended numbers‐at‐risk table and confidence intervals. The chemotherapy KM curve crosses the two experimental arm KM curves, suggesting that the proportional hazards (PH) assumption is implausible. However, Rizvi et al. [20] report hazard ratios, estimated from two separate Cox models for the experimental treatments relative to chemotherapy. We will follow this and assess the extent to which their primary results may have been affected by treatment switching. If they are prespecified, other measures of treatment effect are likely to be considered more appropriate in situations where the PH assumption is violated, and we return to this issue in the discussion. Following Rizvi et al. [20] two separate Cox models that regress on treatment group, stratified by the randomisation stratification factor histologic subtype (squamous or non‐squamous), were used to estimate the two ITT hazard ratios. These regressions resulted in estimated hazard ratios of 0.768 (95% confidence interval from 0.592 to 0.995) for durvalumab relative to chemotherapy and 0.874 (95% confidence interval from 0.677 to 1.129) for durvalumab plus tremelimumab relative to chemotherapy, where a hazard ratio of less than one favours the experimental treatment. Because of the adjustment for two interim analyses, Rizvi et al. present 97.54% and 98.77% confidence intervals. However, our main interest lies in the sensitivity of the point estimates to treatment switching and so we will use 95% confidence intervals throughout.

### 2.2 Switching to Immunotherapy at the First Subsequent Treatment

#### 2.2.1 Control Group Patients

From Figure 1 we can see that 158 patients were randomized to chemotherapy. However, 94 of these control group patients received a subsequent treatment that was considered distinct from their randomised treatment when assessing treatment efficacy. Of these 94 patients, 52 received a form of immunotherapy as part of their first subsequent treatment and 42 did not. Both experimental treatments are a form of immunotherapy. Our motivating example therefore illustrates the common situation where the concern is not that control group patients switch to an experimental treatment, but that they switch to something similar to the experimental group. We can use the same two‐stage treatment switching methodology in this situation where the hypothetical scenario is not that control group patients do not switch to the experimental treatment, but that they do not switch to something that is considered similar to this treatment.

### 2.3 Experimental Group Patients

From Figure 1 we can see that 160+161=321 patients were randomized to one of the experimental treatment groups. Of these patients, 71+54=125 received a subsequent treatment that was considered distinct from their randomised treatment when assessing treatment efficacy. Of these 125 patients, only 2 (both in the durvalumab group) received a form of immunotherapy as part of their first subsequent treatment. In applications where an appreciable proportion of experimental group patients receive immunotherapy in their first switch, and it is plausible that they may receive benefit or harm from switching in this way, then we should also consider hypothetical approaches that require making statistical adjustments for these treatment groups. However, in our application, this is neither necessary nor possible because of the very small number of experimental group patients who receive immunotherapy in their first subsequent treatment. We return to this issue in the discussion.

#### 2.3.1 Terminology: Switching to the ‘Experimental Arm’ Versus ‘Switching to Immunotherapy’

When describing statistical methods it is clearer to refer to control group patients switching to the experimental treatment. In many applications, this will be the type of switching that we wish to address. A complication is that, for the MYSTIC trial, the concern is not that control group patients switch to an experimental treatment, instead there is the concern that they switch to a similar treatment, that is, an immunotherapy. When describing statistical methods (Sections 4 and 5) we will therefore refer to control group patients switching to the experimental treatment. When we apply them to the MYSTIC trial (Section 6) we will refer to them switching to an immunotherapy.

### 2.4 An Alternative Secondary Baseline

As explained in the introduction, our proposal is to use the time of the first subsequent treatment as the secondary baseline. For example, by using this secondary baseline, we will be able to target the estimand where control group patients do not receive immunotherapy in their first subsequent treatment but instead receive another subsequent treatment. We will also be able to target estimands where proportions different from zero subsequently receive immunotherapy (see Section 3 below).

**FIGURE 2:**
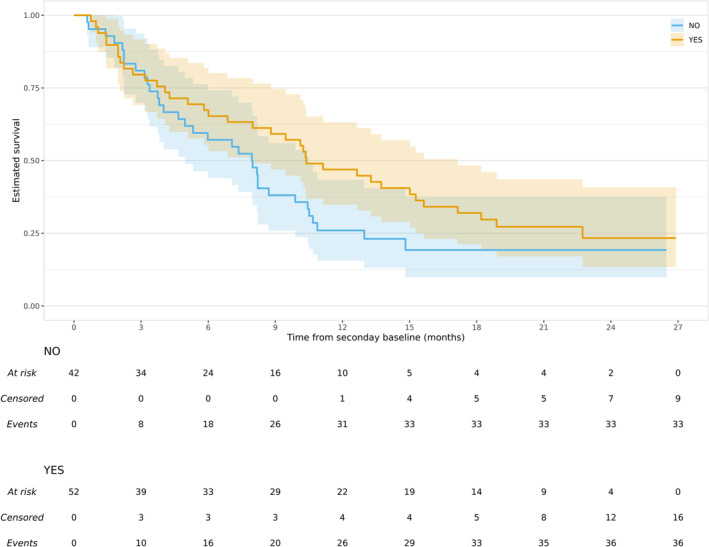
Kaplan–Meier curves for post‐secondary baseline survival times for MYSTIC trial control group patients who receive a subsequent treatment, stratified by whether the first subsequent treatment included immunotherapy. No and yes indicate that the first subsequent treatment did not include, and did include, immunotherapy, respectively.

In Figure 2 we show KM curves comparing the post‐secondary baseline survival times of the 52 control group patients who received immunotherapy in their first treatment switch (YES) to the 42 patients who similarly switched but to other forms of treatment (NO). The KM curves separate after around 3 months but diagnostics (not shown) suggest that models assuming a common treatment effect, such as a Cox or accelerated failure time models, are adequate for these data.

Figure 2 might be taken as evidence that control group switchers benefit from switching to immunotherapy. However, as explained in the introduction, we must adjust for confounding at the secondary baseline; there is no randomisation to justify this unadjusted comparison of the two patient groups in Figure 2.

## 3 Estimands for Our Motivating Example

One of our aims is to show how our proposed two‐stage methods can be incorporated into the estimands framework. In terms of the International Council for Harmonisation (ICH) framework [3], for our motivating example, we use estimands with the following attributes. Population: the MYSTIC trial's primary analysis population of patients with ≥ 25% of tumor cells expressing PD‐L1. The variable (endpoint) is overall survival: the time from patient randomization to death. The treatment conditions of interest are the two experimental treatments and the control treatment, and the population summary for the variable is a pair of hazard ratios comparing the two experimental treatments to the control. The ITT analysis above handles the intercurrent event of treatment switching using a treatment policy strategy.

We will target three alternative estimands (Table 1). We will therefore make inferences in counterfactual worlds where control group patients may receive a different type of treatment (immunotherapy vs. non‐immunotherapy) in their first treatment switch.

**TABLE 1:** Three alternative estimands used in analyses of the MYSTIC trial.

Estimand	Description
A	No control group patients receive immunotherapy in their first subsequent treatment
B	All control group patients, who receive a subsequent treatment, receive immunotherapy in their first subsequent treatment
C	70% of control group patients, who receive a subsequent treatment, receive immunotherapy in their first subsequent treatment

### 3.1 Estimand A

Estimand A is the effect of randomised treatment if no control group patients receive immunotherapy in their first subsequent treatment. This estimand is similar to the more usual one that is targeted by conventional two‐stage analyses, and the RPSFTM, which estimates treatment effects where control group patients never receive the experimental treatment. The key distinctions between estimand A, and this more usual estimand, are that it relates to control group patients not receiving any form of immunotherapy, rather than the two specific types in the two experimental arms, and it allows them to receive immunotherapies at further lines of therapy. Estimand A is very useful but our aim is to use two further estimands to build a fuller narrative about the impact that control group patients subsequently receiving immunotherapy has.

### 3.2 Estimand B

Estimand B is the effect of randomised treatment if all control group patients receiving a subsequent treatment receive immunotherapy in their first treatment switch. Together, estimands A and B represent the two most extreme rates at which control group patients receive immunotherapy. By using estimands A and B we are able to better understand the impact that this particular type of treatment switching has by placing bounds on this.

### 3.3 Estimand C

Finally, estimand C is the effect of randomised treatment if 70% of control group patients who receive a subsequent treatment receive immunotherapy in their first treatment switch, where this percentage is motivated by the real‐world study of Nadler et al. [21]. In their web Supporting Information, Nadler et al. [21] find that in first‐line chemotherapy patients who receive a second‐line treatment, 2048 (monotherapy) + 79 (combined therapy) = 2127 receive immunotherapy in their second line; 636 (further chemotherapy) + 273 (targeted therapy) = 909 do not. Therefore, 70% of first‐line chemotherapy patients receive immunotherapy in their first subsequent treatment. This proportion is greater than the 52/94 = 55% in the MYSTIC trial. By quantifying the benefit of patients receiving the two experimental treatments, compared with control where chemotherapy patients then receive immunotherapy at a realistic rate, estimand C is likely to be of more interest to healthcare decision‐makers. This is because estimand C matches the second‐line therapy mix of control group patients to the rates observed in clinical practice.

Estimation methods for estimand C require further statistical assumptions about the mechanism determining which control patients receive immunotherapy in their first subsequent treatment. Our position is that the definition of estimands should, in general, be free from statistical assumptions, so we describe these modelling assumptions in Section 5.

### 3.4 Experimental Group Patients

Nadler et al. [21] also provide information concerning second‐line treatments for immunotherapy patients, and so treatment switching rates that are relevant to the two experimental arms of the MYSTIC trial. As very few experimental group patients receive immunotherapy in their first treatment switch (Section 2.2), one possible approach is to use a treatment policy strategy for the experimental arms. However, this approach is likely to be objectionable because different strategies are then used across the three treatment groups. Furthermore, this could be erroneously thought to imply that hypothetical strategies are only appropriate for control group patients. Another, and in our view preferable, approach is to use a hypothetical strategy for experimental group patients where the intercurrent events of treatment switching occur at the observed rate. We, therefore, adopt this hypothetical approach throughout. This does not require making statistical adjustments for experimental group patients when performing the estimation.

## 4 Conventional Two‐Stage Estimation

In this section, we describe conventional two‐stage estimation [10, 11] that uses the time of disease progression as the secondary baseline. When using the conventional approach we assume that control group patients may only switch to the experimental treatment when their disease progresses.

Let $n_{\mathrm{cp}}$ be the number of control group patients who have a secondary baseline (i.e., whose disease progresses). Let $I_{i}$, $i=1,2,\cdots ,n_{\mathrm{cp}}$, be an indicator for the *i*th such patient, where $I_{i}=1$ indicates that they switched to the experimental treatment at their secondary baseline and $I_{i}=0$ indicates that they did not switch treatments. Let $T_{i}$ and $S_{i}$, $i=1,2,\cdots n_{\mathrm{cp}}$, be the survival times and the times (from randomisation) to the secondary baseline, respectively. Let $X_{i}$ be the vector of covariates for the *i*th patient, recorded at or before their secondary baseline survival time.

We model the $n_{\mathrm{cp}}$ post‐secondary baseline survival times, $T_{i}^{*}=T_{i}-S_{i}$ using an accelerated failure time model. We therefore assume that $T_{i}^{*}$, $i=1,2,\cdots n_{\mathrm{cp}}$, is a continuous random variable with a survival function

(1) $$P\left(T_{i}^{*}>t|I_{i},X_{i}\right)=S_{\theta }\left(\exp\left(-\alpha I_{i}-{\mathrm{\beta X}}_{i}\right)t\right)$$

where $S_{\theta }\left(t\right)$ is an assumed form for the baseline ($I_{i}=0$ and $X_{i}=0$) survival function; the subscript θ emphasises that model (1) will also include other parameters, for example for the Weibull model the shape and scale. The parameter $\phi =\exp\left(\alpha \right)$ in model (1) is the acceleration factor associated with a control group patient switching to the experimental treatment at their secondary baseline, relative to a similar patient who does not switch treatments. If ϕ>1≡α>0 then switching to the experimental treatment is associated with longer post‐secondary baseline survival times. In model (1) we assume that there is no unmeasured confounding, that is, that all confounders have been measured and adjusted for by including them in $X_{i}$.

The counterfactual (if they had instead not received the experimental treatment at their secondary baseline) survival times $U_{i}$, for the subset of the $n_{\mathrm{cp}}$ control group patients who received the experimental treatment at their secondary baseline, is assumed to be

(2) $$U_{i}=S_{i}+T_{i}^{*}/\phi$$

The rationale for the counterfactual time $U_{i}$ in Equation (2) is that the post‐secondary survival time $T_{i}^{*}$ for control group patients who switch to the experimental treatment at their secondary baseline would instead have been $T_{i}^{*}/\phi$ if they had, counter to fact, not switched treatment. We assume that the effect associated with ϕ in model (1) is causal when computing the counterfactual in Equation (2). The assumption that the counterfactual $U_{i}$ is related to the observed survival times as in Equation (2) is a strong assumption that is needed to justify the two‐stage method. This assumption is made much more plausible by adjusting for confounding when estimating ϕ but in general strong and untestable assumptions will be required when performing statistical analyses that adjust for treatment switching.

We estimate the acceleration factor $\phi =\exp\left(\alpha \right)$, and the other parameters β and θ, in model (1) by fitting accelerated failure time models to the post‐secondary baseline survival times $T_{i}^{*}$, using maximum likelihood and the $n_{\mathrm{cp}}$ control group patients with a secondary baseline. We then substitute $\phi =\hat{\phi }$ into (2) to estimate the counterfactual survival times $U_{i}$ for control group patients who switched to the experimental treatment. We therefore estimate what these patients' survival times would have been if they had instead not switched treatments, and so had remained on their assigned control group treatment throughout the trial. Adjusted analyses (that make inferences in the counterfactual world where control group patients may not receive the experimental treatment) are then performed by replacing the survival times of control group patients who received the experimental treatment at their secondary baseline by their estimated $U_{i}$ and fitting the required analysis models to the resulting adjusted dataset.

### 4.1 Re‐Censoring

One issue with the above approach for performing adjusted analyses is that the censoring mechanism may be uninformative for the observed $T_{i}$ but informative on the counterfactual time scale $U_{i}$. Re‐censoring is a way to ensure that censoring is uninformative on the counterfactual scale, and so guards against bias because of informative censoring in adjusted analyses, but incurs loss of information. This information loss can have more serious implications than are immediately obvious. For example, Latimer et al. [18] point out that re‐censoring can result in a type of ‘missing information bias’ when the treatment effect changes over time. This is because re‐censoring loses information in the longer term. However, for specific datasets, the impact of re‐censoring can be hard to predict. We agree with Latimer et al. [18] that analyses should be performed with and without re‐censoring, so that the sensitivity of the results to this decision can be assessed.

For each patient, we define their potential censoring time $C_{i}$. This is their data cut‐off (DCO) time (the difference between their DCO date and their randomisation date) unless the patient is censored before DCO, in which case $C_{i}$ is their observed censoring time. We should however distinguish between two types of censored patients. For patients censored because they reach their DCO time (i.e., the end of the trial) we therefore take their $C_{i}$ to correspond to their DCO time, which presents no issues. However, for patients censored because they drop out, for example, because they withdraw consent during the trial, further assumptions are required. For example, we may justify using their drop out time instead of their DCO time by assuming that the resulting censoring is uninformative on the observed $T_{i}$ scale. This is because re‐censoring guards against informative censoring on the counterfactual scale $U_{i}$ under the assumption that the censoring is uninformative on the observed $T_{i}$ scale.

We re‐censor all control group patients at their earliest possible counterfactual censoring time, under all possible treatment trajectories and the model for the counterfactual $U_{i}$ in Equation (2) [17]. This time for the *i*th control group patient is $C_{i}/\phi$ if ϕ>1, which is their censoring time under the assumption that they switch to the experimental treatment immediately after randomisation. To accommodate ϕ<1 where the earliest possible counterfactual censoring time for the *i*th control group patient is $C_{i}$, we define $D_{i}=\min\;\left(C_{i},C_{i}/\phi \right)$. Hence $D_{i}=C_{i}$ if ϕ<1, as required. We then re‐censor all control group patients at $D_{i}$: if their $D_{i}<U_{i}$, then $U_{i}$ is replaced by $D_{i}$ and their event indicator is replaced by 0. As in Equation (2) when calculating counterfactual survival times, we substitute $\phi =\hat{\phi }$ in all these calculations. The intuition for re‐censoring is that (assuming censoring at $C_{i}$ is uninformative on the observed $T_{i}$ scale) censoring at the earliest possible time that $C_{i}$ corresponds to, across all counterfactual treatment trajectories, will be uninformative for any treatment trajectory that could occur.

### 4.2 Allowing for the Uncertainty in the Acceleration Factor

There is uncertainty in the estimation of ϕ. The simplest approach is to ignore this and approximate $\phi =\hat{\phi }$ throughout. Here we compute the counterfactual survival times from Equation (2), optionally re‐censor, and then fit the required analysis models to the adjusted dataset where we replace all survival times for control group patients receiving the experimental treatment with their counterfactual survival times. The limitation of this approach is, by ignoring the uncertainty in $\phi =\hat{\phi }$, confidence intervals from the adjusted analyses will be too narrow.

Bootstrapping should be used to compute more accurate confidence intervals but raises issues about the most appropriate type of bootstrapping to use and its computational intensity. This is because a wide variety of methods for bootstrapping are possible, including the parametric and non‐parametric bootstrap, percentile or Wald‐based confidence intervals. We provide details of how bootstrapping can be implemented when using our proposed methodology in Section 5 below.

## 5 Proposed New Methods for Two‐Stage Estimation

We now develop a variety of new methods for two‐stage estimation. We will illustrate these methods using our motivating example in the next section.

### 5.1 Using the Time of First Subsequent Treatment as the Secondary Baseline (Estimands A, B and C)

In this section, we describe how conventional two‐stage estimation is modified to implement our alternative secondary baseline. We redefine some quantities in Section 4 when describing our proposed approach.

Let $n_{\mathrm{cs}}$ be the number of control group patients who receive a subsequent treatment. For our motivating example $n_{\mathrm{cs}}=94$ (Figure 2). Let $I_{i}$, $i=1,2,\cdots ,n_{\mathrm{cs}}$, be an indicator for the *i*th such patient, where $I_{i}=1$ indicates that their first subsequent treatment includes the experimental treatment. For the reasons given in Section 2.3.1, for our motivating example $I_{i}=1$ indicates that the first subsequent treatment includes immunotherapy, and $I_{i}=0$ indicates that the first subsequent treatment does not include immunotherapy. Let $T_{i}$, $i=1,2,\cdots n_{\mathrm{cs}}$, be the survival times and let $S_{i}$ now be the time (from randomisation) to the first subsequent treatment. Hence $T_{i}^{*}=T_{i}-S_{i}$ continues to be the survival times after the secondary baseline, but now the secondary baseline is the time of first subsequent treatment. The variable $I_{i}$ continues to be an indicator for a control group patient receiving the experimental treatment, but now it is defined for the $n_{\mathrm{cs}}$ control group patients who receive a subsequent treatment and relates to patients' first subsequent treatment.

We model the $n_{\mathrm{cs}}$ post‐secondary baseline survival times, $T_{i}^{*}=T_{i}-S_{i}$ (shown in Figure 2 for our motivating example) using an accelerated failure time model as in Equation (1). This model is fitted using maximum likelihood and in the same way as in the conventional approach. The counterfactual (if they had instead not received the experimental treatment in their first subsequent treatment) survival times $U_{i}$, for the subset of the $n_{\mathrm{cs}}$ control group patients who received the experimental treatment in their first subsequent treatment, is then computed as shown in Equation (2), substituting $\phi =\hat{\phi }$, again as in the conventional approach.

We may optionally re‐censor in the way described in Section 4.1 and use some form of bootstrapping to allow for the uncertainty in the acceleration factor (Section 4.2). We will use a non‐parametric percentile‐based bootstrap procedure where 10,000 bootstrap samples are produced and the bootstrap sampling is performed within each treatment group, forcing the same number of patients in each group (but not the same numbers of control group patients who switch treatments) as in the observed data for each bootstrap sample. We then estimate ϕ for each bootstrap sample by fitting an accelerated failure time model. Computing the required counterfactual survival times then produces the final bootstrap sample. By fitting analysis models to each bootstrap sample, the bootstrap replications (e.g., estimated hazard ratios) can be computed. Finally, the 2.5% and 97.5% quantiles of the bootstrap replications can be used to provide 95% confidence intervals.

### 5.2 Propensity Score Methods for Performing the First Stage Estimation (Estimands A, B and C)

Equation (1) is a simple and direct way to adjust for the confounders at the secondary baseline, $X_{i}$. An alternative is to use propensity score adjustment. Here we fit a logistic regression (or another statistical model), to the control group patients who receive a subsequent treatment, to estimate the probability $P\left(I_{i}=1|X_{i}\right)$. Propensity score weighting [19] can then be used. Propensity scores can be used to estimate the average treatment effect (ATE) and the average treatment effect for the treated (ATT) [19]. Here the ATT is more suitable, so that we make inferences for ϕ in the population of control group patients who receive the experimental treatment in their first subsequent treatment (because we require counterfactual survival times for this group of patients). Propensity score weighting has been used previously [14] but our recommendation to use the ATT is a novel feature of our methodology. Propensity score adjustment methods require different types of assumptions to more conventional regression based methods, for example, ‘positivity’, so that $0<P\left(I_{i}=1|X_{i}\right)<1$. Hence using both approaches is a practical way to assess if conclusions are robust to some of the assumptions made when adjusting for confounders. The positivity assumption is in general more plausible when using our proposed secondary baseline than the conventional one. This is because we then compare control group patients who receive different first subsequent treatments instead of patients who did, and did not, switch treatments at disease progression.

We therefore allocate weights of $P\left(I_{i}=1|X_{i}\right)/\left(1-P\left(I_{i}=1|X_{i}\right)\right)$ for $I_{i}=0$ patients when fitting model (1), and weights of one to the $I_{i}=1$ patients, and omit the term ${\mathrm{\beta X}}_{i}$, when estimating $\phi =\exp\left(\alpha \right)$ in model (1) for the post‐secondary baseline survival times (Figure 2). If confidence intervals are presented from these weighted acceleration failure time models then they must be based on robust/sandwich standard errors or bootstrapping. We continue to compute the counterfactual survival times $U_{i}$ from (2) and, if desired, re‐censor as explained in Section 4.1. The propensity score model for $P\left(I_{i}=1|X_{i}\right)$, and the resulting weights, can be estimated for each bootstrap replication (Section 4.2) when using this form of confounder adjustment, to also allow for their uncertainty.

Further modifications are possible, for example, we may standardise the weights for the $I_{i}=0$ patients so that they are on average one when estimating $\phi =\exp\left(\alpha \right)$, so that the $I_{i}=0$ and $I_{i}=1$ groups are ensured to provide a suitable amount of information to the estimation of θ. We could also consider using a method that is doubly robust, or instead use stratification or matching [19] to implement the propensity score adjustment. We will explore the implications of using propensity score weighting to adjust for confounding at the secondary baseline and return to this issue in the discussion.

### 5.3 Non‐Accelerated Failure Time Models and Quantile Matching (Estimands A, B and C)

A key observation is that the methodology for accelerated failure time models in Sections 4 and 5.1 ‘matches the quantile’ of the observed $T_{i}^{*}$ for control group patients who switch to the experimental treatment in their first subsequent treatment when computing the counterfactuals $T_{i}^{*}/\phi$. To see why this is so, for an accelerated failure time model (1) we have

(3) $$P\left(T_{i}^{*}>t|I_{i}=1,X_{i}\right)=S_{\theta }\left(\exp\left(-\alpha \right)\exp\left(-{\mathrm{\beta X}}_{i}\right)t\right)$$

and

(4) $$P\left(T_{i}^{*}>t/\phi |I_{i}=0,X_{i}\right)=S_{\theta }\left(\exp\left(-{\mathrm{\beta X}}_{i}\right)t/\phi \right)=S_{\theta ,{\mathrm{\beta X}}_{i}}\left(t/\phi \right)$$

where, from the definition $\phi =\exp\left(\alpha \right)$, Equation (3) is equal to Equation (4).

A less direct way to compute the counterfactual post‐secondary baseline survival time $T_{i}^{*}/\phi$ in Equation (2) is to first compute Equation (3), for control group patients who switch to the experimental treatment in their first subsequent treatment, where t is replaced by their observed $T_{i}^{*}$ (also replacing θ and β with their estimates). Here we use the survivor function for the model fitted to the post‐secondary survival times and interpret the resulting quantity as the ‘observed quantile’. For example, if the observed quantile is computed to be 0.5 for a particular patient then we conclude that they survived to their median post‐secondary baseline survival time (given that they received the experimental treatment in their subsequent treatment). We then compute $T_{i}^{*}/\phi$ by equating the survivor function in Equation (4) to the observed quantile, again replacing t with the observed $T_{i}^{*}$ (also replacing θ and β with their estimates), and applying the corresponding inverse of the survivor function $S_{\theta ,{\mathrm{\beta X}}_{i}}$ to solve for $T_{i}^{*}/\phi$. For example, we assume, for a patient whose observed quantile is 0.5, that if they had instead not received the experimental treatment then they would have survived to the corresponding median post‐secondary baseline survival time. This is a strong and untestable assumption that is required when using quantile matching but we again note that, in general, strong assumptions will be needed when adjusting for treatment switching.

Re‐censoring is implemented in a similar way by censoring all control group patients at their earliest possible counterfactual censoring time, under all possible treatment trajectories and the model for their counterfactual survival times. Here we equate Equation (3), conditioning on $I_{i}=1$ irrespective of any subsequent treatments received, and Equation (4), both with $t=C_{i}$, and apply the inverse survivor function corresponding to (4) to compute $f_{i}\left(C_{i},\phi \right)=C_{i}/\phi$. We then define $D_{i}=\min\left(C_{i},f_{i}\left(C_{i},\phi \right)\right)$ and re‐censor all control group patients at their $D_{i}$.

The ‘match the quantile’ approach is indirect and unnecessarily complicated when using an accelerated failure time model for the post‐secondary baseline survival times. However, its advantage is that its principles can be used for any survival model. More generally for control group patients, who switch to the experimental treatment in their first subsequent treatment, we compute observed quantiles from the fitted survivor function

(5) $$P\left(T_{i}^{*}>t|I_{i}=1,X_{i}\right)=S\left(t|I_{i}=1,X_{i}\right)$$

where t is replaced by the observed $T_{i}^{*}$ (we suppress the dependence of this calculation on estimated model parameters in Equation (5)). We then assume that these patients would have survived to the same observed quantiles if they had instead not received the experimental treatment in their first subsequent treatment. Hence we equate these observed quantiles to

(6) $$P\left(T_{i}^{*}>t|I_{i}=0,X_{i}\right)=S\left(t|I_{i}=0,X_{i}\right)$$

and solve for t to compute the required counterfactual post‐secondary baseline survival times. The counterfactual post‐secondary survival times are therefore obtained by applying the inverse survivor function corresponding to Equation (6) to the observed quantiles calculated from Equation (5).

Re‐censoring is optionally applied to all control group patients by computing Equation (5) with $t=C_{i}$, and so conditioning on $I_{i}=1$ irrespective of any subsequent treatments received. We then equate the result to Equation (6) and solve for t to obtain the earliest possible counterfactual censoring time $f_{i}\left(C_{i}\right)$. We then define $D_{i}=\min\left(C_{i},f_{i}\left(C_{i}\right)\right)$ and re‐censor all control group patients at their $D_{i}$.

### 5.4 Adjusted Analysis Where All Control Group Patients Who Switch Treatments Receive the Experimental Treatment in Their First Subsequent Treatment (Estimand B)

As explained in Section 3, we will explore a hypothetical estimand B where all control group patients in the MYSTIC trial receiving a subsequent treatment receive immunotherapy in their first treatment switch. As also explained in Section 3, estimands A and B represent the two most extreme rates at which control group patients receive immunotherapy. By using both these estimands we are able to better understand the impact of control group patients subsequently receiving an immunotherapy.

Under an accelerated failure time model for post‐secondary baseline survival times, instead of computing the counterfactual $U_{i}$ in Equation (2) for control group patients who receive the experimental treatment in their first subsequent treatment, we now compute the counterfactual survival time (if they had instead received experimental treatment) for control group patients who did *not* receive experimental treatment in their first subsequent treatment

(7) $$U_{i}=S_{i}+T_{i}^{*}\phi$$

Note that we have redefined $U_{i}$ to define the counterfactual now required. The rationale for the counterfactual time $U_{i}$ in Equation (7) is that the post‐secondary survival time $T_{i}^{*}$ for control group patients who do not switch to the experimental treatment would instead have been $T_{i}^{*}\phi$ if they had, counter to fact, received the experimental treatment in their first treatment switch. If confounders are adjusted for by including them as covariates then the estimates of ϕ in Section 5.1 may be used. However, if propensity score adjustment is used (Section 5.2), then the average treatment effect for the control (ATC) is the most suitable estimand to use, so that we make inferences for ϕ in the population of control group patients who do not receive the experimental treatment in their first subsequent treatment (because we require counterfactual survival times for this group of patients). One way to implement the ATC is simply to use $1-I_{i}$ as the outcome in the propensity score modelling. We now define $D_{i}=\min\;\left(C_{i},C_{i}\phi \right)$ and (if implemented) we re‐censor all control group patients at $D_{i}$. The observation that the ATC is now the most suitable estimand to use when using propensity score adjustment, but the same estimates of ϕ can be used if confounders are adjusted for by including them as covariates, raises the issue of whether conventional regression‐based adjustment is entirely appropriate. We return to this issue in the discussion.

Similarly, the ‘match the quantile’ approach (Section 5.3) may be modified by using Equation (6) as the basis to compute observed quantiles for $I_{i}=0$ control group patients and then Equation (5) as the basis for computing their counterfactual post‐secondary baseline survival times. Alternatively, and perhaps more simply, $1-I_{i}$ could be used instead of $I_{i}$ in all modelling. Bootstrapping may also be used to allow for the uncertainty in ϕ.

### 5.5 Adjusted Analysis Where Different Proportions of Control Group Patients Receive the Experimental Treatment in Their First Subsequent Treatment (Estimand C)

The estimands in Sections 5.1 and 5.4 adopt hypothetical strategies where no control group patients receive the experimental treatment in their first subsequent treatment, and all control group patients who receive a subsequent treatment receive the experimental treatment in their first treatment switch, respectively. In this section, we develop methods to estimate our estimand C where a particular proportion of control group patients in the MYSTIC trial receive immunotherapy in their first switch. Note that when defining estimand C in Section 3 we made no statements about the statistical assumptions or modelling required to estimate this quantity. In particular, the mechanism that determines which control patients receive immunotherapy in their first subsequent treatment was not described.

Our proposed estimation method is a relatively straightforward modification of the estimation methods in Sections 5.1 and 5.4: a random selection of control group patients who receive a subsequent treatment have their observed times to event replaced by their counterfactuals (Equations (2) and (7), when modifying the methods in Sections 5.1 and 5.4, respectively). We modify the method in Section 5.1 if a smaller proportion of control group patients receiving the experimental treatment in their first subsequent treatment is required, and we modify the method in Section 5.4 if a larger proportion is required. These procedures can be justified by assuming that control group patients who receive a subsequent treatment, and hypothetically switch to a different treatment to the one observed, are a random sample of those who received their observed treatment. The simplest possible mechanism that determines which control patients receive immunotherapy is therefore assumed.

For example, if we modify the method in Section 5.1 where only one randomly selected control group patient has their counterfactual (2) computed then the assumed proportion (of those receiving a subsequent treatment) of control group patients who receive the experimental treatment in their first treatment switch is reduced, from its observed proportion, by $1/n_{\mathrm{cs}}$. Similarly, if the method in Section 5.4 is modified where only one randomly selected patient has their counterfactual (7) computed this assumed proportion is increased by $1/n_{\mathrm{cs}}$.

By computing counterfactuals for different numbers of randomly selected patients, when modifying one of the methods in Sections 5.1 and 5.4, we can target estimands that assume any unit fraction, where the denominator is $n_{\mathrm{cs}}$, of control group patients who receive a subsequent treatment receive the experimental treatment in their first switch. This should be adequate to approximate any population proportion required. The quantile matching method could also be used, where a random selection of control group patients who receive a subsequent treatment have their observed times to event replaced by their counterfactuals, so that non‐accelerated failure time models may also be used in conjunction with this approach.

One issue is that different random selections of patients will result in notable differences in adjusted results. To overcome this issue, the process can be repeated many (100, say) times and the resulting adjusted estimates pooled using Rubin's rules [22] for multiple imputations. Here the indicator for which patients we compute counterfactual survival times is conceptualised as the missing data. In principle, bootstrapping (Sections 4.2 and 5.1) could be used to allow for the uncertainty in the estimate of ϕ but using 10,000 bootstrap replications for a method that involves creating many simulated datasets is computationally expensive. A less computationally demanding approach is therefore likely to be desirable. One potential approach is to simulate a different value of ϕ, from the normal approximation used to compute its confidence interval, to use when creating each simulated dataset. This is analogous to using a different random draw of parameters for each imputed dataset when using multiple imputations. Alternatively, the acceleration factor ϕ and the proportion of control group patients that receive the experimental treatment in their first switch could be treated as fixed parameters whose values can be changed so that their implications can be explored. Given the wide variety of estimation methods for ϕ, and the proportions of control group patients that could be considered, some form of sensitivity analysis is perhaps the most prudent approach.

Another issue is that we include confounders in our modelling of post‐secondary baseline survival times. These variables are therefore treated as important predictors of control group patients switching to the experimental treatment. However, their predictive power is not used when randomly selecting patients. More sophisticated approaches may therefore be desirable and we return to this issue in the discussion.

## 6 Illustrating Our Proposed Methods Using the Motivating Example

We now apply our proposed statistical methods (Section 5) using our motivating example (Section 2). As explained in Section 2, we make statistical adjustments for control group patients receiving immunotherapy in their first subsequent treatment, rather than the specific forms of immunotherapies in the two experimental arms of the MYSTIC trial.

### 6.1 Confounding at the Secondary Baseline

A variety of potential confounders at the secondary baseline were identified before investigating their prognostic power: sex, age, baseline body mass index (BMI), baseline ECOG status, liver metastasis at baseline (Yes/No), geographical region (Asia, Europe, North America), histologic subtype (squamous or non‐squamous), current smoking status (Yes/No), progression by secondary baseline (Yes/No), the number of serious adverse events by the secondary baseline and the log time to secondary baseline. Race was also considered as a potential confounder but was highly correlated with geographical region and whether control group patients subsequently received immunotherapy was thought more likely to depend on geographical region than race. The log time to the secondary baseline was thought to be a potentially useful surrogate for other post‐baseline events that occur by the secondary baseline.

First, stepwise Cox regression was used to determine which potential confounders appear to be predictive of outcome. Here Cox regressions were sequentially fitted to the 94 ‘control group switchers’ post‐secondary baseline survival times (Figure 2) where an indicator for the patient receiving immunotherapy in their first treatment switch was included in all models. Forwards and backwards AIC selection both resulted in the selection of four covariates: geographical region, age, liver metastasis at baseline and log time to secondary baseline. Second, stepwise logistic regression was used to determine which potential confounders appear to be predictive of exposure. Here logistic regressions were sequentially fitted to the same 94 control group patients where the outcome was a binary variable indicating that the first subsequent treatment included immunotherapy. Forwards selection resulted in the selection of two variables: geographical region and baseline ECOG. Backwards selection resulted in the selection of five variables: geographical region, baseline ECOG, sex, age and current smoking status.

One approach, that we also considered and would have adopted had it been feasible, is to adjust for any covariate that is identified as predictive of either outcome or exposure. However, the number of covariates selected is then large, given the relatively small sample size of 94 control group patients with post‐secondary baseline survival times (Figure 2), and propensity score balance diagnostics indicated that we could not successfully adjust for so many covariates. Instead of attempting to adjust for the union of covariates that were selected as either predictive of outcome or exposure, we instead adjusted for their intersection. VanderWeele [23] calls this the ‘common cause’ approach [24]. We, therefore, adjust for geographical region and age in all analyses of post‐secondary baseline survival times. The confounder adjustment used here is feasible, given the sample size, but is best regarded as pragmatic. We return to this issue in the discussion.

### 6.2 Estimand A: Analyses Using Accelerated Failure Time Models

To explore the sensitivity to the choice of the accelerated failure time model used for post‐secondary baseline survival times, four possibilities were considered: the exponential, the Weibull, the lognormal and loglogistic models. All four of these models are implemented by the survreg function provided by the survival package in R, which was used to produce adjusted analyses. These four models were considered sufficient to explore this sensitivity and we return to this issue in the discussion.

Analyses were performed with and without re‐censoring (Section 4.1), using both propensity score adjustment for confounders (Section 5.2, where the weights for $I_{i}=0$ patients were standardised so they are on average one) and including confounders in the regression model (Equation (1)). In total therefore we considered 4 models  × 2 methods for adjusting for confounding = 8 estimates of ϕ. With two conventions for re‐censoring, this results in 16 adjusted analyses. As explained in Section 5.1, non‐parametric bootstrapping was used to take into account the uncertainty in ϕ.

The estimated acceleration factor $\hat{\phi }$ was sensitive to the method used for confounder adjustment but not the choice of parametric survival model (Table 2). To two decimal places, across all eight models $\hat{\phi }$ took values in the range 1.25–1.70 (Table 2). For comparison, using propensity score adjustment to estimate the ATE (instead of the ATT as shown), the four estimated acceleration factors were 1.490, 1.482, 1.488 and 1.565. These estimates are much closer to the $\hat{\phi }$ obtained by including the confounders in the regression model, suggesting that the differences between the estimated acceleration factors using propensity score adjustment may be because of the fact that the ATT targets a different population to more conventional, regression‐based, adjustment.

**TABLE 2:** Properties of eight accelerated failure time models fitted to the post‐baseline survival times of control group patients who receive a subsequent treatment in the MYSTIC trial (Figure 2).

Model	Method for confounder adjustment	$\hat{\phi }$	AIC
Exponential	Inclusion as covariate (Equation (1))	1.609 (0.992, 2.610)	966.2
Weibull	Inclusion as covariate (Equation (1))	1.584 (1.022, 2.453)	967.2
Lognormal	Inclusion as covariate (Equation (1))	1.636 (0.993, 2.697)	960.9
Loglogistic	Inclusion as covariate (Equation (1))	1.702 (1.058, 2.736)	959.5
Exponential	Propensity score weighting (Section 3.1.2)	1.256 (0.762, 2.070)	939.6
Weibull	Propensity score weighting (Section 3.1.2)	1.256 (0.762, 2.070)	941.6
Lognormal	Propensity score weighting (Section 3.1.2)	1.250 (0.743, 2.101)	935.1
Loglogistic	Propensity score weighting (Section 3.1.2)	1.301 (0.764, 2.214)	936.9

*Note:* Estimates of the acceleration factor ϕ are followed by 95% confidence intervals in parentheses, where robust standard errors are used for propensity score adjustment. Note that AIC statistics from models that include confounders as a covariate and those that use propensity score weighting are not comparable.

**FIGURE 3:**
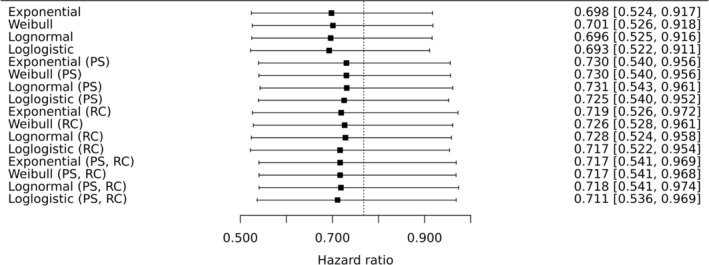
Sixteen adjusted hazard ratios (estimand A) comparing durvalumab (experimental treatment) to chemotherapy (control). PS in parentheses indicates that propensity score adjustment was used to adjust for confounding at the secondary baseline, otherwise, adjustment was made by including confounders as covariates. RC in parentheses indicates that re‐censoring was performed, otherwise this was not performed. All hazard ratios are estimated using cox regression on the treatment group and the stratification factor histologic subtype. Confidence intervals were computed using bootstrapping. The ITT estimated hazard ratio of 0.768 is shown as a vertical line, for comparison.

**FIGURE 4:**
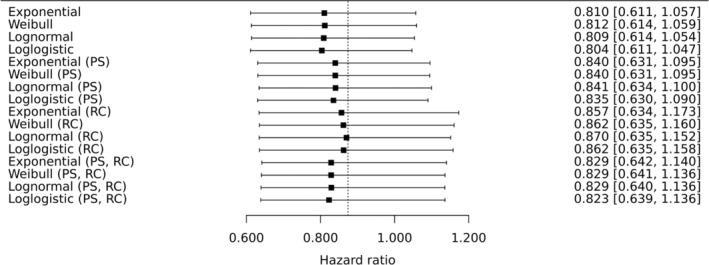
Sixteen adjusted hazard ratios (estimand A) comparing durvalumab plus tremelimumab (experimental treatment) to chemotherapy (control). PS in parentheses indicates that propensity score adjustment was used to adjust for confounding at the secondary baseline, otherwise, adjustment was made by including confounders as covariates. RC in parentheses indicates that re‐censoring was performed, otherwise this was not performed. All hazard ratios are estimated using cox regression on the treatment group and the stratification factor histologic subtype. Confidence intervals were computed using bootstrapping. The ITT estimated hazard ratio of 0.874 is shown as a vertical line, for comparison.

The acceleration factors in Table 2 result in slightly different adjusted hazard ratios comparing durvalumab relative to chemotherapy (Figure 3) and durvalumab plus tremelimumab relative to chemotherapy (Figure 4). If re‐censoring is not applied then larger $\hat{\phi }$ result in more adjustment, and so smaller estimated hazard ratios, as expected. However, the impact of re‐censoring varies from one estimate of ϕ to the next, confirming that the implications of re‐censoring can be hard to predict (Section 4.1). Perhaps most notably, re‐censoring reduces adjustments made by the largest values of $\hat{\phi }$. This is likely subject to the type of ‘missing information bias’ discussed in Section 4.1. This is because from Figure 1 we can see that the effects of the experimental treatments become evident quite late in the trial. The overall impression is that the occurrence of control group patients receiving immunotherapy in their first subsequent treatment has likely diluted the treatment policy hazard ratios, estimated in the ITT analysis (Section 2.1), for both comparisons at the second decimal place.

Analyses that ignore the uncertainty in ϕ, and take confidence intervals directly from standard software when fitting analysis models to adjusted datasets, resulted in slightly narrower confidence intervals. For example, if we take the analysis where the loglogistic model was used, confounders were adjusted for by including them as covariates and re‐censoring was not performed, the resulting bootstrap 95% confidence intervals were (0.522, 0.911) (Figure 3) and (0.611, 1.047) (Figure 4). Instead ignoring the uncertainty in ϕ resulted in 95% confidence intervals of (0.533, 0.901) and (0.621, 1.041). The adjusted analyses for the MYSTIC trial are not very sensitive to the estimate of ϕ (Table 2 and Figures 3 and 4) so allowing for its uncertainty is not necessarily crucial. However, this would seem to be worthwhile and may be more important for other examples.

### 6.3 Estimand A: Analysis Using Non‐Accelerated Failure Time Models and Quantile Matching

To illustrate the use of quantile matching (Section 5.3) we applied a Gompertz (proportional hazards) survival model to the post‐secondary baseline survival times (Figure 2) using the flexsurvreg [15] R package. We adjusted for the confounders region and age by including them as covariates, via the term $\beta X_{i}$ in the linear predictor, similar to the accelerated failure time model (1). We also included the effect of receiving immunotherapy, with the associated log hazard ratio α.

The ‘pgompertz’ (Gompertz cumulative distribution) function from the flexsurvreg package was used to compute the observed quantiles (Equation (5)) from which the counterfactual post‐secondary baseline survival times were computed using the ‘qgompertz’ (Gompertz inverse cumulative distribution or quantile function). These counterfactual survival times were then added to the $S_{i}$ to produce counterfactual times to event for $I_{i}=1$ patients. This procedure resulted in estimated hazard ratios of 0.696 and 0.809 for durvalumab, and durvalumab plus tremelimumab, relative to chemotherapy, respectively. These results are in good agreement with the covariate‐adjusted results using accelerated failure time models (first four results are shown in Figures 3 and 4). Re‐censoring was also optionally applied, resulting in estimated hazard ratios of 0.725 and 0.870 for durvalumab, and durvalumab plus tremelimumab, relative to chemotherapy, respectively. These results are also in good agreement with the covariate‐adjusted results using accelerated failure time models (ninth to twelfth results shown in Figures 3 and 4). Based on its AIC statistic of AIC = 968.1, this model is a worse fit than the accelerated failure time models considered in Section 3 but this additional analysis reassures us that the adjusted results obtained there are robust to the use of an accelerated failure time model for the post‐secondary baseline survival times.

Given its poor AIC statistic and similar adjusted hazard ratios, this Gompertz model was not pursued further. However, it could be combined with bootstrapping to provide confidence intervals that allow for model uncertainty for the secondary baseline survival times and/or used in conjunction with propensity scores to adjust for confounding.

### 6.4 Estimands A, B and C: Supplementary Estimands Exploring the Implications of Different Proportions of Control Group Patients Receiving the Experimental Treatment in Their First Subsequent Treatment

We illustrate the use of the methods in Sections 5.4 and 5.5 where ϕ, and the proportion of control group patients who receive immunotherapy in their first subsequent treatment, are treated as fixed parameters. These parameters may then be changed, so that their implications can be explored. A very wide range of possible parameter combinations are available, and so we restrict ourselves to providing some indicative results.

We will explore the implications of four different proportions of control group patients, who receive a subsequent treatment, receiving immunotherapy in their first treatment switch. We apply the method in Section 5.5 (using 100 replications and Rubin's rules), where 14 control group patients who received a non‐immunotherapy first subsequent treatment are randomly selected to receive immunotherapy in their first switch, to target estimand C. Then we have (52 + 14)/94 = 70% MYSTIC patients switching treatments in this way, providing the proportion of 70% of Nadler et al. [21]. We, therefore, compute the required counterfactual (Equation (7)) for 14 randomly selected control group patients. To consider other proportions of control group patients, we also implement the methods described in Sections 5.1 and 5.4 to examine the extremities where these proportions are 0% (estimand A) and 100% (estimand B), respectively. Finally, we interpret the treatment policy estimand, estimated from the ITT analysis, as corresponding to 52/94 = 55% of control group patients who receive a subsequent treatment receiving immunotherapy in their first subsequent treatment, and embed this primary analysis into our framework.

We also need to determine suitable values of ϕ to explore. We have observed that results are not very sensitive to ϕ (Figures 3 and 4), and so for illustrative purposes, we will restrict our attention to ϕ=1.702. This is the point estimate from the loglogistic model where confounders are adjusted by including them as covariates. This model has the smallest AIC statistic (Table 2) of the four models that used this more conventional form of adjustment. However in general it will be of interest to explore use of alternative values of ϕ. If the implications of a particular two‐stage analysis are to be explored then the uncertainty in the corresponding estimate of ϕ (or the parameters in the model used for post‐secondary baseline survival times if quantile matching is used) should be taken into account, for example, by using bootstrapping.

**FIGURE 5:**
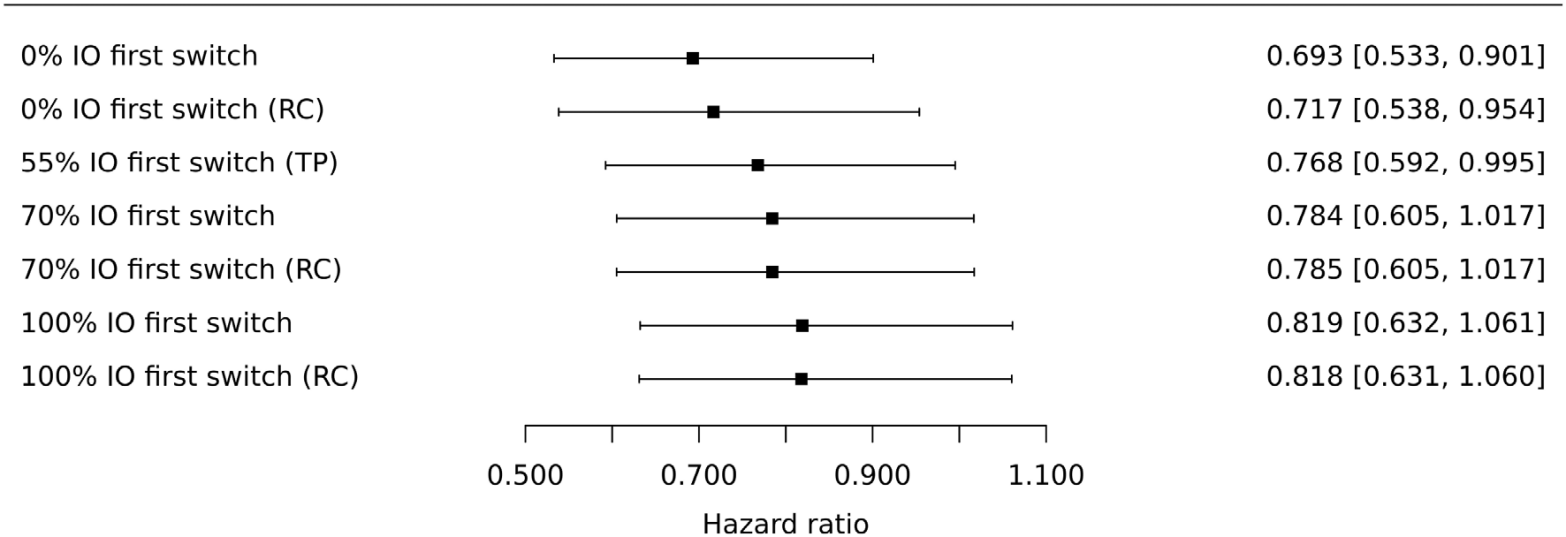
Comparison of durvalumab (experimental treatment) to chemotherapy (control), exploring how the estimated treatment effect depends on the proportion of chemotherapy patients having a subsequent treatment who receive immunotherapy in their first treatment switch. RC in parentheses indicates that re‐censoring was performed, otherwise this was not performed. TP in parentheses indicates that the treatment policy estimand is presented, estimated from the ITT analysis, where 52/94 = 55% of control group patients having a subsequent treatment receive immunotherapy in their first switch. The other percentages use hypothetical strategies for the intercurrent event where control group patients switch treatments: 0%, Section 5.1 (estimand A); 70%, Section 5.5 (estimand C); 100%, Section 5.4 (estimand B). The acceleration factor was taken to be ϕ=1.702.

**FIGURE 6:**
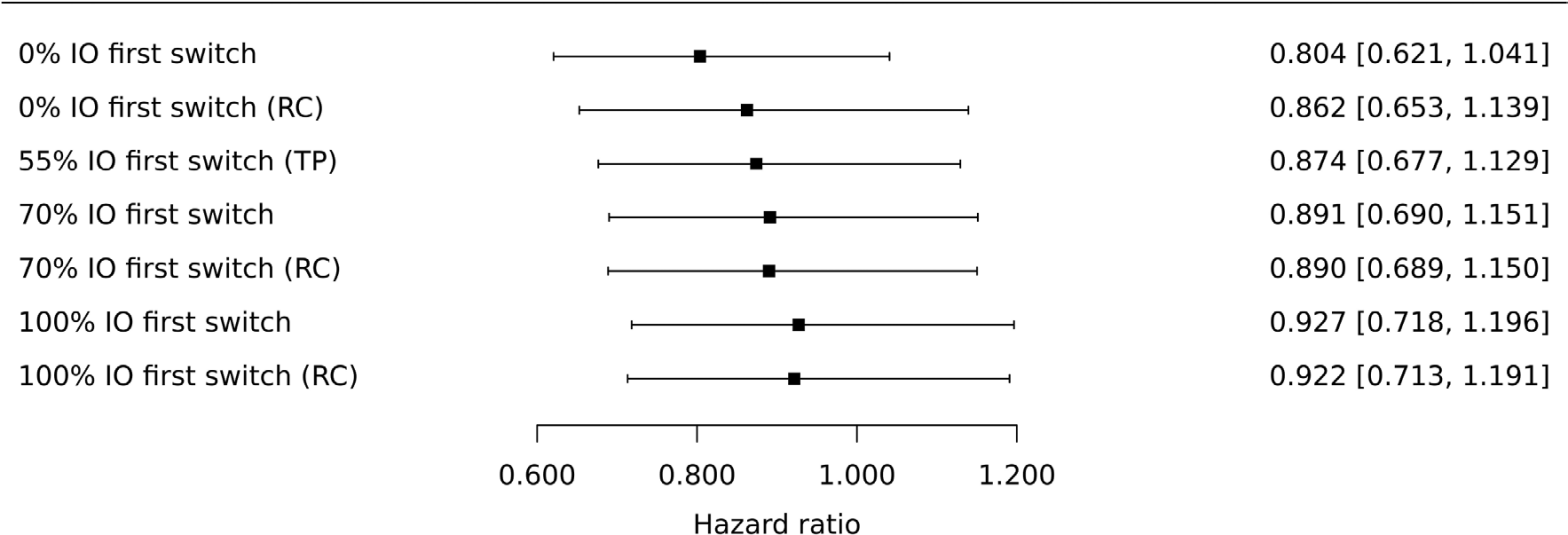
Comparison of durvalumab plus tremelimumab (experimental treatment) to chemotherapy (control), exploring how the estimated treatment effect depends on the proportion of chemotherapy patients having a subsequent treatment who receive immunotherapy in their first treatment switch. RC in parentheses indicates that re‐censoring was performed, otherwise this was not performed. TP in parentheses indicates that the treatment policy estimand is presented, estimated from the ITT analysis, where 52/94 = 55% of control group patients having a subsequent treatment receive immunotherapy in their first switch. The other percentages use hypothetical strategies for the intercurrent event where control group patients switch treatments: 0%, Section 5.1 (estimand A); 70%, Section 5.5 (estimand C); 100%, Section 5.4 (estimand B). The acceleration factor was taken to be ϕ=1.702.

The results are shown in Figures 5 and 6, for the durvalumab and durvalumab plus tremelimumab comparisons with chemotherapy, respectively. RC in parentheses indicates that re‐censoring was performed, otherwise this was not performed. The point estimates where no control group patients receive immunotherapy in their first subsequent treatment are the same as in Figures 3 and 4 for the loglogistic model where regression adjustments are used. This is because the corresponding estimate of ϕ is used as the fixed parameter value.

The overall impression is that the treatment effects are somewhat sensitive to the assumed proportion of chemotherapy patients who receive immunotherapy in their first treatment switch. This is because Figures 5 and 6 indicate that the effect associated with initially receiving the two experimental treatments is notably reduced in populations where chemotherapy patients are likely to receive immunotherapies as their first subsequent therapy. This is an important conclusion for healthcare decision‐makers for universal health care systems, for example, those who must determine if novel therapies are to be available as first‐line or subsequent therapies. This is because the Nadler et al. [21] real‐world study's subsequent treatment trajectories may be more representative of treatments subsequently received by chemotherapy patients in universal healthcare systems.

## 7 Discussion

Our three estimands used for the MYSTIC trial adopted hypothetical approaches, where different proportions of control group patients, who receive a subsequent treatment, receive an immunotherapy in their first treatment switch. Together they build a narrative about the impact that switching to immunotherapies has on the estimated treatment effects. Estimand C is intended to be most relevant to decision‐makers in universal health care systems because it describes the benefit of the experimental treatment where chemotherapy patients may subsequently receive immunotherapy, and so benefit from the form of experimental treatment, at a realistic rate in their next line of therapy. An important issue when defining estimands is that they must be estimable, and here we define estimands where two‐stage estimation may be used. Estimand C is especially novel and further methodological work to better understand the implications of this type of hypothetical strategy would be welcome. We have used estimands that do not require making statistical adjustments for experimental group patients, primarily because so few of these patients receive immunotherapy in their first subsequent treatment. As immunotherapy is often assumed to potentially be curative, it is, in any case, debatable whether or not we would wish to consider hypothetical estimands where different proportions of experimental group patients receive subsequent immunotherapies. This is because experimental group patients may be thought to have received the majority of their potential to benefit from immunotherapy at their initial treatment. However, in similar applications where sufficient numbers of experimental group patients receive immunotherapy in their first subsequent treatment, and it is plausible that they may have benefitted from this, we suggest that it would be prudent to also adopt hypothetical strategies, and so perform statistical adjustments, in the experimental arms. This would be performed in the same way as illustrated for the MYSTIC control group patients.

We have shown that a richer narrative about the implications of treatment switching is possible using our new estimands and associated estimators. We have made proposals regarding our novel use of propensity score methods to adjust for confounding and shown how they can be used in practice. Propensity score adjustment is an alternative to more conventional regression‐based adjustment that targets marginal estimands of post‐baseline treatment effects. The implications of its use may, therefore, be more pronounced when the effect measure used is not collapsible, for example when using a proportional hazards model to estimate a hazard ratio. However, once estimates of post‐baseline treatment effects have been obtained, the same adjustment methods are proposed when using both propensity score and regression‐based adjustment. We, therefore, suggest that determining which of these two methods is used is an issue for estimation, rather than when defining estimands. For the MYSTIC trial, we conclude that treatment policy estimands have been slightly diluted by control group patients subsequently receiving immunotherapy and that they would likely have been further diluted if the extent of this type of switching had been greater.

We followed Rizvi et al. [20] by computing adjusted hazard ratios for the MYSTIC trial but here the proportional hazards assumption is highly dubious. One advantage of our methods is that they can easily accommodate alternatives, that is, non‐proportional hazards, analysis models. A theoretical objection to our proposals is that we have not required that the methods used for computing counterfactual times, and the analysis time to event model, make the same assumptions. A helpful consequence of this pragmatism is that we can then analyse the adjusted datasets in the way best suited to the types of statistical inferences that are required. However, developing methods where the same assumptions are made in both steps would be an interesting development.

One complication of the two‐stage approach is that a very wide variety of methods are available to the analyst. For example, we must determine appropriate models for the post‐secondary baseline survival times, identify confounders and decide upon the statistical method used to adjust for them. We did not attempt to exhaust these possibilities when applying our methods to our motivating example. Hence the results presented are merely intended to give an indication of what is possible. In particular, our stepwise methods for determining which confounders to adjust are pragmatic, and motivated by the fact that our sample is relatively small. In larger datasets, we would propose using more sophisticated statistical methods when identifying and modelling confounders. Sensitivity analyses to the choice of potential confounders included in the analysis will also often be desirable, but even then concerns about residual confounding are likely to persist. In practice, the most important caveat is likely to be that adjusted analyses assume confounding at the secondary baseline has been adequately addressed. A potential disadvantage of our proposed method is that, by comparing only control group patients who receive subsequent treatment, the number of patients in this comparison may be limited. Another disadvantage is we assume that control group patients switch to two distinct forms of treatment.

Further work could compare the use of propensity scores to more conventional regression‐based confounder adjustment, for example in a simulation study. If all related post‐secondary baseline treatment effect heterogeneity is explained by covariates used in the modelling then conventional regression adjustment would seem to be perfectly adequate. However, if there is unexplained heterogeneity of this type then using propensity scores may be preferable. We suspect in practice this heterogeneity will usually be present, and so we cautiously recommend using propensity scores for confounder adjustment when using two‐stage estimation. The observations that the ATT and ATE may substantively differ, and that theory suggests the ATT is more appropriate, are important findings. A simulation study that explores the implications of using different propensity score methods for confounder adjustment is required to investigate further.

Further work could also develop more sophisticated estimation methods when targeting estimands where a particular proportion of patients receive the experimental treatment. By randomly selecting patients, our estimates do not reflect the fact that some patients may be more likely to receive the experimental treatment than others. The development of more sophisticated estimation methods, that both reflect this and enforce the required proportion, would be worthwhile. For example, one possibility might be to ‘clone’ control group patients whose subsequent treatment may be changed to create two individuals: one with subsequent treatment changed and one with treatment unchanged, with suitable weights. Valid standard errors could then be calculated using bootstrapping. More sophisticated methods that assume there are more distinct groups of treatment would also be valuable. The type of statistical adjustment for treatment switching we have discussed would not be necessary where observed subsequent treatments are very similar to those of the target estimand. Other methods would be needed to perform other types of statistical adjustment, for example, population adjustment, if required.

Quantile matching could replace the conventional scaling of survival times using accelerated failure time models. This is because it accommodates this standard method and enables us to use many other statistical models for post‐secondary baseline survival times. However, we recommend caution in this regard because quantile matching is implied by an accelerated failure time model rather than intrinsically being the conventional assumption. However quantile matching could in principle be used for any post‐secondary baseline survival model, for example, this could include mixture models, flexible spline‐based models, and so on. In most trials, enthusiasm for sophisticated survival modelling is likely to be dampened by the sample size that is available but for large trials, more ambitious modelling is facilitated by quantile matching. We have followed the conventional approach by applying parametric survival models to the post‐secondary baseline survival times but an alternative is to use an RPSFTM g‐computation approach to estimate the acceleration factor using these data. Provided that the statistical test underlying this g‐computation adequately adjusts for confounders, the resulting estimated acceleration factor could then be used to compute adjusted survival times, and so perform adjusted analyses, in a ‘hybrid RPSFTM two‐stage’ analysis. This proposal is worthy of further investigation, for example, in simulation studies, and may form the subject of future work.

Our proposed secondary baseline of the time of first subsequent treatment makes two‐stage estimation widely applicable to situations where patients need not switch treatment when their disease progresses. Other alternative secondary baselines may be possible. Another tantalizing secondary baseline is the time of receiving the experimental treatment for the first time, for control group patients who subsequently receive this in any subsequent treatment and the time to first subsequent treatment for other control group patients. Then, by comparing the post‐secondary baseline survival times, we can attempt to remove all experimental treatment received by control group patients in two‐stage adjusted analyses. However, this choice of secondary baseline raises immediate concerns because whether a switch to a non‐experimental treatment is used as the secondary baseline depends on whether or not the patient prospectively receives the experimental treatment. Simulation studies could be used to determine if this concern is a serious source of bias in practice.

To conclude, we have developed a variety of new estimands and estimation methods for two‐stage estimation. Our new estimands allow us to build a more extensive narrative about the implications of treatment switching and our estimation methods allow us to use a wider class of models and methods for adjusting for confounding. We have illustrated our new ideas using a recent trial where a non‐negligible proportion of control group patients receive immunotherapy, which is of the same class as the experimental treatment. We hope others will consider using our proposal in their applications and motivate further methodological work.

## Conflicts of Interest

Dan Jackson, Di Ran, Fanni Zhang, Mario Ouwens, Vitaly Druker, Michael Sweeting and Robert Hettle are employed by AstraZeneca.

## Data Availability

The data in this article were provided by AstraZeneca. Research data are not shared.
